# E‐cigarette use among gender and sexuality diverse (LGBTQA+) adolescents in Australia: The case for LGBTQA+ affirmative harm reduction

**DOI:** 10.1111/dar.14052

**Published:** 2025-05-18

**Authors:** Sasha Bailey, Emma L. Barrett, Scarlett Smout, Lucinda Grummitt, Lyra Egan, Lauren Gardner, Emily A. Stockings, Maree Teesson, Yael Perry, Nicola C. Newton

**Affiliations:** ^1^ The Matilda Centre for Research in Mental Health and Substance Use, Faculty of Medicine and Health The University of Sydney Sydney Australia; ^2^ Telethon Kids Institute The University of Western Australia Perth Australia

**Keywords:** adolescents, alcohol, gender, mental health, school, sexuality, vaping

## Abstract

**Introduction:**

This study aimed to provide a first‐ever comprehensive epidemiology of vaping behaviours among Australian gender and sexuality diverse (LGBTQA+) youth.

**Methods:**

Using cross‐sectional data from the Health4Life study, (*N* = 4,445 students, Mage = 15.7yrs), descriptive statistics and hierarchical mixed effects logistic regression models with nested random intercepts were used to calculate prevalence and differences in vaping behaviours by gender (trans [*n* = 142] vs. cisgender [*n* = 4,144]) and sexuality (gay or lesbian [*n* = 77], bisexual [*n* = 279], questioning [*n* = 167], queer [*n* = 90] vs. heterosexual [*n* = 3,638]), and associations of vaping with use of cigarettes and alcohol (including binge drinking), depression, and anxiety.

**Results:**

Over one‐third of trans and bisexual adolescents had ever tried vaping. Trans adolescents were significantly more likely to report ever vaping, daily vaping, and intention to vape in the future, compared with their cisgender peers, irrespective of age, socio‐economic status, and school. Relative to heterosexual peers, gay/lesbian adolescents were nearly three times as likely to report current regular use of vaping and bisexual adolescents were significantly more likely to report ever vaping and intentions to vape in the future. Among LGBTQA+ adolescents, ever using a vape was associated with increased odds of ever using cigarettes, ever binge drinking, ever drinking alcohol alone, probable depressive/anxiety disorders.

**Discussion:**

Vaping is significantly more common among LGBTQA+ adolescents, especially trans and bisexual adolescents, compared with their cisgender, heterosexual peers. Government health and education bodies should partner with LGBTQA+ community organisations to address the unique contexts of vaping among LGBTQA+ young people in an LGBTQA+ affirmative harm reduction manner.

## 1 INTRODUCTION

Use of e‐cigarettes (vaping) during adolescence poses several unintended and unwanted potential harms to physical and mental health [1, 2]. Emerging studies have quantified the burden of vaping among general population Australian adolescents to inform prevention and intervention efforts [3, 4]. A recent cross‐sectional survey found that over one in four school‐aged adolescents in the general Australian population reported ever using a vape [3]. However, limited research is available regarding patterns and impacts of vaping among gender and sexuality diverse (including those who identify with lesbian, gay, bisexual, trans, non‐binary, queer, questioning, asexual or agender communities; henceforth respectfully referred to using the umbrella term, ‘LGBTQA+’) adolescents [5]. This warrants urgent attention because prior research shows LGBTQA+ young people use cigarettes more frequently, at higher intensities, and at earlier ages, and experience significantly higher rates of depression and anxiety, compared with their cisgender and heterosexual peers [5, 6, 7, 8, 9, 10, 11, 12, 13].

Increasing evidence deriving from young people in the general population also demonstrates a strong interaction between vaping and mental ill‐health [2], with many young people vaping to assuage higher levels of perceived stress [14] and anxiety [15]. Much of the existing literature, and indeed Australia‐specific literature, does not consider the unique needs of LGBTQA+ young people in vaping harm reduction efforts. Anti‐vaping campaigns are often perceived as unrelatable and uninteresting by LGBTQA+ young people [16]. Moreover, though there is mixed evidence regarding the strength and clinical significance of any association between gender affirming hormones (GAHT) and cardiovascular disease risk and venous thromboembolism, vaping may further compound these risks among trans young people using GAHT [17]. Moreover, though there is a paucity of research discerning the precise, clinically significant elevated risk of cardiovascular disease associated with GAHT, vaping may add to this risk among trans young people. High‐quality epidemiological evidence regarding vaping among LGBTQA+ adolescents is needed to ensure the meaningful inclusion of LGBTQA+ adolescents in vaping prevention health strategy and policy planning activities.

To address these gaps, we estimated prevalence and disparities in patterns and onset of e‐cigarette use by gender (trans [participants with a gender different to the gender presumed for them at birth] vs. cisgender [participants with a gender congruent with their gender presumed at birth]) and sexuality (gay or lesbian, bisexual, questioning, and queer vs. heterosexual). We also examined associations between ever using a vape and associated harms, including ever using a cigarette (cigarette uptake), ever binge drinking, solitary alcohol use, depression and anxiety.

## 2 METHODS

### 2.1 Study design and participants

Participants were adolescents aged 14–17 years (*N* = 4445; *M*_age_ = 15.7 years) attending 70 schools across New South Wales, Queensland, and Western Australia between 1 July and 31 December 2022, who completed the 36‐month post‐baseline follow‐up survey of the Health4Life cluster randomised controlled trial (67% of 6640 baseline participants) [18, 19, 20]. Baseline schools were only approached if the relevant ethics approval had been obtained and were required to have a minimum of 30 year 7 students (aged 11–13 years) and were recruited across four geographical sites: Greater Sydney (New South Wales); regional areas of New South Wales; a 100 km radius from Brisbane (Queensland); and a 600 km radius from Perth (Western Australia). All year 7 students who were fluent in English and attending participating schools in 2019 (baseline) were eligible. This 36‐month follow‐up cross‐sectional timepoint was chosen for the present study because it was the only available timepoint that included measures of vaping patterns. Data were collected via self‐report online or hardcopy questionnaires during class.

### 2.2 Measures

*Gender*: Participants were asked one item regarding sex assigned at birth (options included ‘Male’, ‘Female’ or ‘Prefer not to answer’) and one item regarding gender (options included ‘Male’, ‘Female’, ‘Non‐binary’, ‘I use a different term (please specify)’ and ‘Prefer not to answer’). Participants who responded with a gender different to their sex assigned at birth were coded as ‘Trans’ and those with a gender congruent with their sex assigned at birth were coded as ‘Cisgender’. Participants open‐ended responses to ‘I use a different term’ were double‐screened by two researchers (S.B. and S.S.). Valid responses (determined by whether the response alluded to a verifiable trans community) were coded as ‘Trans’ and invalid responses were excluded from these analyses. Hence, trans participants included trans women, trans men, non‐binary people and other gender diverse people as identified within open‐text responses. Those responding ‘Prefer not to say’ for the gender item were excluded for these analyses.

*Sexuality*: Participants were asked about their sexuality via one item with options, including ‘Straight (heterosexual)’, ‘Gay or lesbian’, ‘Bisexual’, ‘I use a different term (please specify)’, ‘Don't know’ or ‘Prefer not to say’. Participants responding ‘Gay or lesbian’ were coded as being Gay or Lesbian, and those responding Bisexual were coded as being Bisexual. Participants open‐ended responses to ‘I use a different term’ were again double‐screened by two researchers (S.B. and S.S.) with valid responses coded as ‘Queer’ and invalid (mock) responses excluded from these analyses. Participants responding ‘Don't know’ were coded as Questioning. Participants responding ‘Prefer not to say’ were excluded from the present analyses. Heterosexual participants were the referent group for sexuality‐level analyses.

*Vaping*: A single yes/no item assessed whether participants had ever used a vape (‘Have you ever used an e‐cigarette, even one or two puffs?’). A definition and common terms for vaping were also provided. A separate item assessed participants' age of first using a vape (‘At what age did you first use an e‐cigarette?’). Participants who responded ‘Age 10 or below’, ‘Age 11’, ‘Age 12’, or ‘Age 13’ were coded as initiating vaping ‘before age 14’ and those who responded ‘Age 14’, ‘Age 15’, ‘Age 16’ and ‘Age 17 or above’ were coded as initiating vaping ‘at or after age 14 years’. Vaping before age 14 was of interest because previous studies have shown that substance use before age 14 poses markedly higher risks of short‐ and long‐term unintended consequences, including school disengagement, substance use harms and dependence, and experiences of mental ill‐health [21]. Furthermore, 14 years of age was selected as the point of dichotomy for vaping initiation in this study because the mean age of onset of vaping in this sample (as reported in a previous study) was found to be 14.0 [5]. Thus this age point provides the best distinction between those who initiate “early”, that is, before the mean age (<14) and those who are “average initiators” who start at or beyond the mean age (>14).

One item assessed recent use (‘When did you last use e‐cigarettes?’) with possible options, including 0 = ‘In the past day/24 hours’, 1 = ‘More than 1 day ago, but within the past 7 days’, 2 = ‘More than 7 days ago, but within the past 30 days’, 3 = ‘More than 1 month ago, but within the past 3 months’, 4 = ‘More than 3 months ago, but within the past 6 months’, 5 = ‘More than 6 months ago, but within the past 12 months’, and 6 = ‘More than 12 months ago’. Those responding 0, 1, 2 or 3 were coded as having used a vape within the past 3 months, whereas participants responding 0, 1 or 2 were coded as having used a vape within the past month. An additional item regarding frequency/intensity of vaping use (‘How frequently did/do you use e‐cigarettes?’) was also used for these analyses, with possible options, including 0 = “Just tried them once, haven't used again”, 1 = “Once a month or less”, 2 = “Three or more days in the past month”, 3 = “Weekly”, and 4 = “Daily”. From these items, two dichotomous (yes/no) categorical variables were derived to reflect intensity: vaping during the past month (those responding ‘0’, ‘1’ and ‘2’) and vaping during the past 3 months (‘0’, ‘1’, ‘2’, and ‘3’). A ‘current regular user’ variable was also computed wherein cases were classified as participants reporting past 30 day use and at least weekly frequency of use, aligning with previous research on patterns of vaping use among Australian adolescents [3].

Lastly, a single item assessed participants' intention to use a vape, ‘If your friend offered you a vape, how likely would you be to try it?’. Participants responding ‘Certain to try’ and ‘Likely/very likely’ were coded as ‘Likely to try’, and those responding ‘Undecided’, ‘Very unlikely/unlike’ and ‘Certain not to try’ were coded as ‘Not likely to try’. Undecided was included in the ‘not likely’ group as this is the more conservative view and it is aligned with population level data that the majority of young people do not vape. That is, if a young person nominated ‘undecided’ it is *more* likely they ultimately will not vape than they will. This decision also meant that the “likely” grouping only included those people who made a definitive response of being likely to vape.

*Cigarette use*: Participants were asked a single item assessing ever use of a tobacco cigarette: (‘Have you ever tried smoking a cigarette, even one or two puffs?’). Those who responded ‘yes’ were coded as ‘having ever used a cigarette’, whereas those who responded ‘no’ were coded as ‘not having ever used a cigarette’.

*Alcohol binge drinking and solitary alcohol use*: One yes/no item assessed participants' lifetime experience of binge drinking: ‘Have you ever consumed 5 or more standard alcohol drinks (see chart above) on one occasion?’ Valid cases of lifetime binge drinking were required to have also indicated ever using alcohol. Participants were also asked about solitary drinking: ‘Do you ever use alcohol while you are by yourself, or alone?’ (0 = No, 1 = Yes). To promote accurate responses, students were also shown a standard drinks chart to guide responses. While standard drinks are not universal, standard drinks were defined as 10 g of alcohol aligning with Australian standards [22].

*Depressive disorder*: The Patient Health Questionnaire modified for adolescents (PHQ‐A) was used to assess depressive symptomatology, with one item pertaining to suicidal ideation removed during ethical board review [23]. Participants were asked to indicate how often they had been bothered by eight different symptoms during the past 2 weeks (e.g., ‘Feeling tired, or having little energy?’) with potential responses ranging from 0 (Not at all) to 3 (Nearly every day). Scores were summed and dichotomised. Scores ≥10 denoted probable depressive disorder and scores <9 representing absence of probable depressive disorder [24]. The eight‐item PHQ‐A has demonstrated equivalency of total score correlations and the predictive accuracy to screen depressive symptomatology of the nine‐item version of the PHQ tool [25].

### 2.3 Anxiety disorder

The 13‐item Patient‐Reported Outcomes Measurement Information System for Anxiety among Adolescents aged 11–17 years (PROMIS‐A) was administered to assess participants' levels of anxiety [26]. Participants reported the past two‐week frequency by which they experienced different symptoms and feelings of anxiety such as fear, anxious misery and hyperarousal (e.g., ‘I got scared really easily’). Participants could choose a response between 1 to 5 (1 = Never, 5 = Almost Always) with scores summed and ranging between 13 to 65, with higher scores indicating higher levels of anxiety symptoms. Raw scores were prorated if 75% of items are answered and converted to a T‐score which is further classified into levels of anxiety severity based on previously published norms [27]. Previous item‐specific analyses of the PROMIS‐A questionnaire has demonstrated sound psychometric validity among adolescents aged 11–17 years [26].

### 2.4 Analyses

We calculated proportions of participants who reported using e‐cigarettes ever, during the past 3 months, and during the past month, being a current regular user of vapes, and likelihood of using a vape in the future. Odds ratios were computed from hierarchical logistic regression models with nested random intercepts to control for school‐level clustering to test disparities in patterns of vaping use by gender (trans vs. cisgender) and sexuality (gay or lesbian, bisexual, questioning, and queer vs. heterosexual). To test associations between vaping ever use and substance use (cigarette ever use, binge drinking ever, and solitary alcohol use ever) and mental health (probable depressive/anxiety disorders) specific to LGBTQA+ young people, hierarchical logistic regression models with nested random intercepts were used within a subset of LGBTQA+ participants who were either sexuality diverse and/or trans and not heterosexual or cisgender. These models were selected to reflect study design features, namely that participants reported repeated measures and, moreover, participants were nested within schools. Conflating gender and sexuality is generally avoided in the literature [28]. However, there is significant overlap between gender and sexuality diverse communities wherein many trans people also identify as sexuality diverse [28]. Moreover, gender and sexuality diverse adolescents similarly engage in substance use for altruistic, social, and LGBTQA+ community seeking reasons [29, 30]. Hence, limitations of this approach were balanced against limitations associated with alternatively running separate models, which in turn would produce wide confidence intervals and spurious results. This study was a cross‐sectional complete case analysis and hence did not utilise any imputation for missing cells. Only participants who provided valid gender and/or sexuality data were included in the corresponding gender and sexuality sub‐group analyses. All models included age and socio‐economic status (Family Affluence Scale III) [31] as covariates (in addition to adjusting for school‐level clustering). For mixed effects models with random intercepts, the default sandwich estimator employed by the [glmer] package when fitting a generalised mixed model was used, that is, the standard estimator based on the inverse of the Fisher information matrix. All analyses were conducted using R Version 3.2.3.

## 3 RESULTS

In total, 4435 participants (*M*_age_ = 15.7) completed the Health4Life 36‐month post‐baseline follow‐up survey. Among these participants, 4251 provided sexuality data, of whom 3638 (85.6%) identified as heterosexual, 77 identified as gay or lesbian, 279 (6.6%) bisexual, 167 (3.9%) unsure/questioning, and 90 (2.1%) queer. In total, 4286 participants provided gender data, of whom 4144 (96.7%) participants were cisgender and 142 (3.3%) were trans (binary and non‐binary). In total, data from 4241 participants was used to derive the agglomerated LGBTQA+ sub‐set, of whom there were 3562 (84%) heterosexual participants and 679 (16%) LGBTQA+ participants. Attrition analyses of the Health4Life study by demographic characteristics and primary outcomes have been previously conducted and are detailed elsewhere [20]. Importantly, trans participants were more likely to drop out following baseline compared with cisgender participants. Disproportionate attrition rates affecting trans participants are commonly noted in the literature, underscoring the importance of trans‐affirming, agile methods of follow‐up, including the option for one to easily update their name, the use of gender‐neutral language, and maintenance of privacy and confidentiality [29]. The number of trans participants at baseline, however, was small (*n* = 30) and, moreover, in line with best practice, data regarding participants' genders was drawn from the latest timepoint available (36‐month post‐baseline) [29]. The discrepancy between baseline and 36‐month post‐baseline responses regarding gender (trans vs. cis) can be explained in how trans young people undergo important developmental identity milestones, including recognising and accepting that they are trans through adolescence, that is, more young people feel comfortable sharing that they are trans as time goes on.

### 3.1 Gender and sexuality disparities in vaping use, intensity and intentions

The highest prevalence of ever using a vape was reported among bisexual and trans adolescents (36.5% and 35.3%, respectively). The majority of gay or lesbian adolescents and questioning adolescents reported initiating vaping at or before the age of 14 years (77.3% and 74.2%, respectively).

Trans adolescents were significantly more likely to report ever vaping (adjusted odds ratio [aOR] 1.59; 95% confidence interval [CI] 1.09, 2.33) and a likely intention to use vapes in the future (aOR 1.73; 95% CI 1.11, 2.68), compared with their cisgender peers. Gay or lesbian adolescents were nearly three times as likely to report being current regular vape users (aOR 2.96; 95% CI 1.10, 7.95) compared with their heterosexual peers. Bisexual adolescents were significantly more likely to report ever using a vape (aOR 1.64; 95% CI 1.25, 2.16) and significantly more likely to report intending to use a vape in the future (aOR 1.46; 95% CI 1.05, 2.03) compared with their heterosexual counterparts, but no other sexuality diverse groups showed significant differences to their heterosexual peers. Questioning adolescents were significantly less likely to report vaping in the past month (aOR 0.41; 95% CI 0.17, 0.99) compared with their heterosexual comparators. All models adjusted for age, socio‐economic status, and school‐level clustering. Full results are detailed below in Tables 1, 2.

**TABLE 1:** Prevalence of vaping use, age of initiation, intensity and intention to use among gender and sexuality diverse emerging adults.

	Gender (*n* = 4286)		Sexuality (*n* = 4251)				
	Trans men, trans women and non‐binary people (*n* = 142)	Cisgender men and women (*n* = 4144)	Gay or lesbian (*n* = 77)	Bisexual (*n* = 279)	Questioning (*n* = 167)	Queer (*n* = 90)	Heterosexual (*n* = 3638)
	*N* (%)						
Vaping ever use	47 (35.3)	1007 (25.6)	22 (28.9)	99 (36.5)	32 (20.3)	19 (22.1)	890 (25.9)
Vaping initiation ≤14 years of age	28 (60.9)	28 (59.9)	17 (77.3)	53 (53.0)	23 (74.2)	9 (47.4)	537 (60.2)
Vaping use—past 3 months	28 (21.2)	495 (12.6)	12 (16.0)	53 (19.6)	10 (6.3)	13 (14.9)	438 (15.6)
Vaping use—past 1 month	28 (21.1)	495 (21.1)	12 (16.0)	53 (19.6)	10 (6.3)	13 (14.9)	438 (12.8)
Vaping intensity—weekly	18 (13.5)	249 (6.3)	9 (12.0)	25 (9.2)	6 (3.8)	6 (6.9)	223 (6.5)
Vaping intensity—daily	15 (11.3)	168 (4.3)	7 (9.3)	17 (6.3)	6 (3.8)	5 (5.7)	152 (4.4)
Current regular user of vaping	22 (16.8)	317 (8.3)	12 (16.4)	33 (12.4)	6 (3.8)	8 (9.6)	285 (8.5)
Vaping intention to use—likely	29 (21.8)	529 (13.5)	10 (13.3)	54 (19.9)	20 (12.7)	14 (16.3)	465 (13.6)

**TABLE 2:** Logistic mixed effects models investigating differences in patterns of vaping among gender‐ and sexuality‐diverse adolescents accounting for differences in age and socio‐economic status.

	Vaping—ever use	Vaping initiation ≤14 years of age	Vaping—past 3‐months	Vaping—past month	Vaping intensity—daily	Current regular user of vapes	Likely intention to use vapes in future
Trans^a^	**1.59** * **(1.09, 2.33)**	0.94 (0.50, 1.76)	1.42 (0.76, 2.64)	1.30 (0.70, 2.41)	**2.25** * **(1.16, 4.36)**	1.65 (0.87, 0.35)	**1.73** ** **(1.11, 2.68)**
Gay or Lesbian^b^	1.05 (0.62, 1.79)	2.03 (0.72, 5.71)	1.27 (0.51, 3.16)	2.04 (0.82, 5.08)	2.14 (0.82, 5.62)	**2.96** * **(1.10, 7.95)**	0.94 (0.47, 1.87)
Bisexual^b^	**1.64** *** **(1.25, 2.16)**	0.71 (0.46, 1.09)	1.06, (0.68, 1.64)	0.82 (0.52, 1.29)	1.00 (0.57, 1.75)	0.96 (0.60, 1.53)	**1.46** * **(1.05, 2.03)**
Questioning^b^	0.72 (0.47, 1.08)	1.81 (0.79, 4.19)	0.42 (0.19, 0.94)	**0.41** * **(0.17, 0.99)**	1.08 (0.43, 2.75)	0.42 (0.16, 1.06)	0.81 (0.49, 1.35)
Queer^b^	0.84 (0.50, 1.44)	0.56 (0.22, 1.43)	1.74 (0.66, 4.61)	1.71 (0.67, 4.38)	1.78 (0.62, 5.14)	1.68 (0.60, 4.68)	1.28 (0.71, 2.31)

*Note*: All models adjusted for age, socio‐economic status, and school‐level clustering. Bold values indicate statistically significant results.

^a^ Compared to cisgender participants as the reference group.

^b^ Compared to heterosexual participants as the reference group.

* *p*< 0.005.

** *p* < 0.01.

*** *p* < 0.001.

**TABLE 3:** Logistic mixed effects models with nested random intercept testing associations between vaping ever use and cigarette uptake and hazardous drinking among gender and sexuality diverse adolescents (*n* = 679) compared to cisgender, heterosexual peers (*n* = 3562).

	Cigarette—ever use	Binge drinking ever	Alcohol use alone ever	Probable depressive disorder	Probable anxiety disorder
	aOR 95% CI				
Vape—ever use	**2.14** * (**1.18**, **3.89**)	**3.98** *** (**3.06**, **5.19**)	**6.19** *** (**4.87**, **7.87**)	**1.96** *** (**1.64**, **2.35**)	**1.87** *** (**1.55**, **2.26**)

*Note*: All models adjusted for age, socio‐economic status, and school‐level clustering. Bold values indicate statistically significant results.

* *p* < 0.05.

*** *p* < 0.001.

### 3.2 Associations between ever using a vape and mental health and substance use outcomes among gender and sexuality diverse adolescents

Among LGBTQA+ adolescents, ever using a vape was associated with increased odds of ever using a cigarette (aOR 2.14; 95% CI 1.18, 3.89), ever binge drinking (aOR 3.98; 95% CI 3.06, 5.19), ever using alcohol alone (aOR 6.19; 95% CI 4.87, 7.87), probable depressive disorder (aOR 1.96; 95% CI 1.64, 2.35) and probable anxiety disorder (aOR 1.87; 95% CI 1.55, 2.26), regardless of age, socio‐economic status, and school. Full model results are displayed in Table 3.

## 4 DISCUSSION

To our knowledge, these findings represent the first epidemiological insights into patterns of vaping among LGBTQA+ adolescents in Australia. Our study highlights the need to consider the unique contexts in which vaping is experienced by LGBTQA+ adolescents to optimally inform meaningful inclusion in vaping prevention efforts.

Our study found that LGBTQA+ young people were twice as likely to report regular, daily vaping, with the highest risk among trans and bisexual adolescents. Higher intensities of vaping were most common among trans adolescents, as were rates of current regular vaping use and intentions to vape in the future. There is limited research to corroborate these findings with, however, the *Writing Themselves In 4* survey of 6418 LGBTQA+ young people in Australia (conducted in 2019) found that approximately 4% of LGBTQA+ young people aged 14 to 17 years are current vape users [5]. Among young people in the general population, the prevalence of vaping ever use affects around one in four young people [3]. Our study suggests vaping is at least double the rate among LGBTQA+ young people compared with cisgender, heterosexual young people in the general population and is increasing in recent years. The exponential increase in rates of vaping use observed in the Australian general population may explain the large discrepancy between vaping prevalence observed in *Writing Themselves In 4* (conducted in 2019) and the present study (conducted in 2022). For example, a recent study found that current use of vaping among young females has increased from 2.4% in 2019 to 20% in 2022–2023 [32]. Nonetheless, this study highlights the importance of the consistent inclusion of adequate indicators [33] of gender and sexuality in large studies of adolescents' patterns of mental health and substance use to allow continued monitoring and agenda‐setting for addressing vaping harms among LGBTQA+ young people.

The present study found that among all gender and sexuality diverse sub‐communities examined, trans adolescents showed the highest prevalence of vaping. Over one in three (35.3%) trans participants reported ever using a vape. While vaping in and of itself does not constitute vaping‐related harms [1], there is a chronic paucity of research examining potential interactions, including adverse interactions, between nicotine‐containing vapes and use of GAHT, such as oestradiol, progesterone, and testosterone [34]. For example, nicotine consumption is associated with an increased risk of thromboembolic events, which can be further exacerbated by oestrogen therapy [35]. Further research is required to understand the interactions between vaping and HRT to identify and prevent potential unintended, unwanted outcomes. Conversely, in the absence of data related to any vaping‐related harms and costs experienced by LGBTQA+ adolescents, especially trans adolescents, future public health efforts should prioritise the promotion of protective factors previously linked with reduced use of vaping. For example, among LGBTQA+ adolescents, this might include establishing and promoting LGBTQA+ affirmative school environments and social supports [36], and for trans adolescents specifically, this may include affordable, timely, and safe access to gender affirming care [35, 37, 38] and legal document gender‐marker change processes [17, 39, 40].

Our findings show that vaping among LGBTQA+ adolescents is strongly correlated with cigarette use, binge drinking, solitary alcohol use, depression, and anxiety, regardless of age, socio‐economic, and school differences. Though these are cross‐sectional associations and preclude inference regarding directionality, these findings illustrate the complex interconnectedness of the use of vaping and the mental health and well‐being of LGBTQA+ young people. Further research is required to understand the motives and expectations of vaping among LGBTQA+ young people, particularly those who are also experiencing comorbid depression and anxiety. Previous research shows that substance use among LGBTQA+ communities is commonly engaged in for altruistic, self‐exploration, and community‐seeking reasons [29, 30]. Future research is required to understand patterns of vaping alongside co‐use of cigarettes, alcohol, and other drugs, and the motivations and expectations of vaping in this unique context of polysubstance use. Our findings highlight the need for further synergy between LGBTQA+ community‐controlled health organisations and local health jurisdictions to better understand the motives and social contexts underpinning vaping use among LGBTQA+ adolescents. Through understanding how and why vaping takes place among LGBTQA+ adolescents, community health workers, educators, and parents will be better placed to refine prevention efforts using an LGBTQA+ affirmative harm reduction manner.

Our findings should be interpreted in light of several limitations. Due to sample size, there was insufficient statistical power to conduct further analyses of LGBTQA+ young people who were both gender and sexuality diverse and who typically experience compounding ill‐health disparities proportionate to their increased levels of minority [12]. This study utilised a non‐probability‐based sample of school‐based adolescents. Though the sample size of LGBTQA+ young people in this sample is sufficiently robust for the present analyses and in alignment with past estimates of the number of LGBTQA+ young people in the Australian general population [13, 41], it is important to note that this sample is not population‐based. Notwithstanding, population‐based datasets tend to underrepresent LGBTQA+ young people and hence many calls in the literature exist highlighting the dual importance of probability‐based and non‐probability‐based datasets when examining the epidemiology of LGBTQA+ youth health. Due to the use of varied gender and sexuality indicators through the Health4Life study, it is difficult to assess patterns in drop‐out among LGBTQA+ participants, who have historically been disproportionately affected by loss to follow‐up and attrition due to issues related to incorrect names/pronouns, privacy issues, and mistrust [29]. It should be noted that the decision to dichotomise mental health measures meant the present study was unable to provide insights into the (continuously measured) severity of mental health outcomes associated with vaping behaviours. Lastly, due to the self‐report, cross‐sectional nature of the study, these findings cannot draw conclusions regarding the directionality of vaping and mental health among LGBTQA+ young people, underscoring the need for future LGBTQA+ targeted longitudinal research.

In summation, uptake and regular use of vaping is significantly higher among LGBTQA+ adolescents, especially trans and bisexual adolescents, compared with heterosexual and cisgender peers, and is associated with alcohol use and mental ill health. Further gender and sexuality inclusive public health surveillance is required to monitor these trends using consistent, adequate indicators of gender and sexuality throughout the study period [29]. Partnerships between LGBTQA+ community‐controlled health organisations and health and education departments are required to understand and address the unique motives, expectations, and contexts of vaping among LGBTQA+ young people in an LGBTQA+ affirmative harm reduction manner.

## AUTHOR CONTRIBUTIONS

SB led the conceptualisation, data curation, formal analysis, methodology, writing – original draft preparation, and writing – review & editing. EB and NN led supervision of SB. EB, NN, SS, LG, LE, LG, EAS, MT, and YP contributed to writing – review & editing. NN, MT, and LG were responsible for funding acquisition and resources for overarching umbrella study from which data for present study derives from.

## FUNDING INFORMATION

Paul Ramsay Foundation, the Australian National Health and Medical Research Council, the Australian Government Department of Health and Aged Care, and the US National Institutes of Health.

## CONFLICT OF INTEREST STATEMENT

The authors declare no conflicts of interest.

## Data Availability

The data that support the findings of this study are available from the Health4Life Chief Investigators and team upon reasonable request.
